# Multiple In Vitro and In Vivo Regulatory Effects of Budesonide in CD4+ T Lymphocyte Subpopulations of Allergic Asthmatics

**DOI:** 10.1371/journal.pone.0048816

**Published:** 2012-12-12

**Authors:** Elisabetta Pace, Caterina Di Sano, Stefania La Grutta, Maria Ferraro, Giuseppe Albeggiani, Giuseppe Liotta, Serena Di Vincenzo, Carina Gabriela Uasuf, Jean Bousquet, Mark Gjomarkaj

**Affiliations:** 1 Istituto di Biomedicina e Immunologia Molecolare, Unità di Immunopatologia e Farmacologia Clinica e Sperimentale dell'Apparato Respiratorio, Consiglio Nazionale delle Ricerche, Palermo, Italy; 2 Environmental Health, ARPA, Palermo, Italy; 3 University Hospital, Montpellier, France; Ludwig-Maximilians-University Munich, Germany

## Abstract

**Background:**

Increased activation and increased survival of T lymphocytes characterise bronchial asthma.

**Objectives:**

In this study the effect of budesonide on T cell survival, on inducible co-stimulator T cells (ICOS), on Foxp3 and on IL-10 molecules in T lymphocyte sub-populations was assessed.

**Methods:**

Cell survival (by annexin V binding) and ICOS in total lymphocytes, in CD4+/CD25+ and in CD4+/CD25- and Foxp3 and IL-10 in CD4+/CD25+ and in CD4+/CD25-cells was evaluated, by cytofluorimetric analysis, in mild intermittent asthmatics (n = 19) and in controls (n = 15). Allergen induced T lymphocyte proliferation and the in vivo effects of budesonide in mild persistent asthmatics (n = 6) were also explored.

**Results:**

Foxp3 was reduced in CD4+/CD25- and in CD4+/CD25+ cells and ICOS was reduced in CD4+/CD25+ cells but it was increased in CD4+CD25-in asthmatics when compared to controls. In asthmatics, *in vitro*, budesonide was able to: 1) increase annexin V binding and to reduce ICOS in total lymphocytes; 2) increase annexin V binding and Foxp3 and to reduce ICOS in CD4+/CD25- cells; 3) reduce annexin V binding and to increase IL-10 and ICOS in CD4+/CD25+ cells; 4) reduce cell allergen induced proliferation. *In vivo*, budesonide increased ICOS in CD4+/CD25+ while it increased Foxp3 and IL-10 in CD4+/CD25+ and in CD4+/CD25- cells.

**Conclusions:**

Budesonide modulates T cell survival, ICOS, Foxp3 and IL-10 molecules differently in T lymphocyte sub-populations. The findings provided shed light on new mechanisms by which corticosteroids, drugs widely used for the clinical management of bronchial asthma, control T lymphocyte activation.

## Introduction

Asthma is a heterogeneous disorder that is characterized by variable and largely reversible airflow obstruction, airway inflammation and hyperresponsiveness. Airway inflammation in allergic asthma is characterized by exaggerated activation of T helper type-2 (Th2) cells, IgE production and eosinophilia [1]. An increased survival of immune effector cells (eosinophils, macrophages and T lymphocytes) within the airways contributes to the severity of asthma [2]. T-lymphocytes play a crucial role in the development of airway inflammation. Activation, differentiation and effector cell function of T-helper cells (Th) are directed by co-stimulatory molecules which deliver critical signals modulating the antigen-specific signal of the T-cell receptor (TCR). Inducible co-stimulatory antigen (ICOS) is one of the most intensively studied co-stimulatory molecules [3]. Emerging evidences in animal models demonstrate a relevant role of ICOS in asthma. Blockade of ICOS in sensitised mice significantly reduced signs of allergic airway inflammation, such as increased IgE and Th2 cytokine production [3] and ICOS-positive regulatory T cells (Tregs) suppress IL-17 production and thereby reverse established, IL-17-dependent airway hyper-reactivity in mice [4]. Tregs exert an important role in the control of T-cell-mediated inflammation in asthma [5] [6]. Two major subsets of Tregs are identified: CD25+ forhead box P3 (Foxp3)+ Tregs and IL-10-producing Tregs. The numbers or function of both Treg subsets are deficient in patients with atopic allergic diseases including asthma [7].

Corticosteroids are the most effective anti-inflammatory agents and topical corticosteroids including budesonide are the recommended therapy in current guidelines for asthma [8]. Corticosteroids act on Tregs, increasing IL-10 production [9] and enhancing IL-10 Treg function [7]. Corticosteroids inhibit T-cell activation and production of Th2 cytokines [10] [11] and increase T cell apoptosis [12] [13].

The objectives of this study were to assess the effects of budesonide (BUD), a potent inhaled corticosteroid, in cell survival and in the expression of ICOS, IL-10 and of Foxp3 in CD4+CD25+ and CD4+CD25- peripheral T lymphocytes obtained from asthmatic patients in comparison to control subjects.

## Materials and Methods

### Subjects and study design

The study was approached by *in vitro* and *in vivo* assessments of BUD effects in asthma. For the *in vitro* evaluations, we selected 19 atopic patients with mild intermittent asthma, according to the criteria of the American Thoracic Society [14], and 15 control subjects without allergic diseases or asthma (Table 1). All asthmatic patients (Table 1) were characterized by a reversible airway obstruction assessed by an increase of ≥12% of forced expiratory volume in one second (FEV_1_) after inhalation of 200 µg of salbutamol. The asthma diagnosis and the assessment of its severity were performed according to the Global Initiative for Asthma [15]. All recruited subjects were never-smokers. Atopy was established by aeroallergen skin prick test (Alk Abellò, Hørsholm, Denmark). None of the patients recruited for evaluating the *in vitro* effects of BUD received any corticosteroid treatment. For assessing *in vivo* effects of BUD, eight atopic steroid naïve patients with mild persistent asthma (Table 1) and uncontrolled disease as assessed by Asthma Control Test (ACT)) (score ≤19) were evaluated before and after 12 weeks of inhaled BUD treatment (twice daily treatment with 200 µg BUD). Pulmonary functional tests (Polgar reference values) and clinical assessment (morning and evening peak expiratory flow (PEF) and ACT were performed before and after BUD treatment. Subjects who had bronchial or respiratory tract infections during the month before the test were not included. The study fulfilled the criteria of the Ethics Committee of Policlinico-Giaccone Hospital-Palermo, was approved and was in agreement with Helsinki Declaration. All subjects had given their written informed consent.

**Table 1:** Demographic and clinical characteristics of the study population.

	Controls = 15	Mild intermittent asthmatics = 19	Mild persistent asthmatics = 8	P value
Caucasic race	15	19	8	
Gender (M/F)	8/7	8/11	4/4	n.s.
Age (years) 50 (25–75) Percentiles	38 (26–45)	27 (19–37)	32.5 (27.5–36.5)	n.s.
FEV1 % of predicted Percentiles	112 (108–119)	99 (93–108)	90.5 (85.5–95.5)	n.s.
FEV1 (PRE), L Percentiles	3.8 (2.97–4.07)	3.40 (2.9–3.9)	3.45 (3.05–4.2)	n.s
FEV1 (POST), L Percentiles	-	3.76 (3.25–4.34)	4.12 (3.44–4.74)	n.s.
Bronchodilation test- Reversibility (%) Percentiles	-	15 (13–16)	15 (13.5–15.5)	n.s.
Parietaria judaica+	-	7/19	3/8	
Olea+	-	4/19	2/8	
Cat+	-	3/19	1/8	
Dermatophagoides Pteronyssinus+	-	8/19	4/8	
Dermatophagoides Farinae +	-	8/19	4/8	
CD4+ cells (%)	44.3±4	50.5±6*	51.7±4.5*	<0.05
CD4+/CD25+ cells (%)	3.5±0.7	4.4±1.6	5±1.9	n.s.
CD4/CD25- cells (%)	40.8±4	46±6*	46.4±3.6*	<0.05

* Mann Whitney versus controls.

### Peripheral blood mononuclear cells (PBMC) cultures

Peripheral blood mononuclear cells (PBMC) were isolated from blood samples (10 ml) of asthmatic patients (mild intermittent and persistent) and of controls by Ficoll-Hypaque (Pharmacia) gradient centrifugation. The cells were suspended in RPMI 1640 tissue culture medium (Invitrogen Life Technologies) supplemented with 1% heat-inactivated FCS (Invitrogen Life Technologies), 2 mM L-glutamine, 20 mM HEPES, 100 U/ml penicillin, 100 µg/ml streptomycin, 5×10^−5^ M 2-ME and 85 µg/ml gentamicin. Purity and viability were tested using trypan blue exclusion.

For assessing *in vitro* effects of BUD, the cells (2×10^6^ cells/ml) were stimulated within tubes (Becton Dickinson, Mountain View, CA) for 24 hours in the absence and in the presence of BUD (Italchimici, Italy) (10^−8^ M final concentration). The concentration range of BUD and incubation times were selected in preliminary experiments (figure S1). Initially, three concentrations of BUD (10^−7^, 10^−8^ and 10^−9^ M) and two time points (24 and 48 hrs) in cell apoptosis preliminary experiments in total lymphocytes were tested. Since 10^−7^ and 10^−8^ M were similar in their effects and were more potent than 10^−9^ M and since the higher effect was observed at 24 hours, the concentration of 10^−8^ M and the time point 24 hours were selected (see figure S1). In some experiments, the cells (2×10^6^ cells/ml) were cultured with/without BUD (10^−8^ M for 24 hours) and then stimulated with the allergen to which the patient was more responsive (for additional 72 hours).

### Flow-cytometry

**Figure 1:**
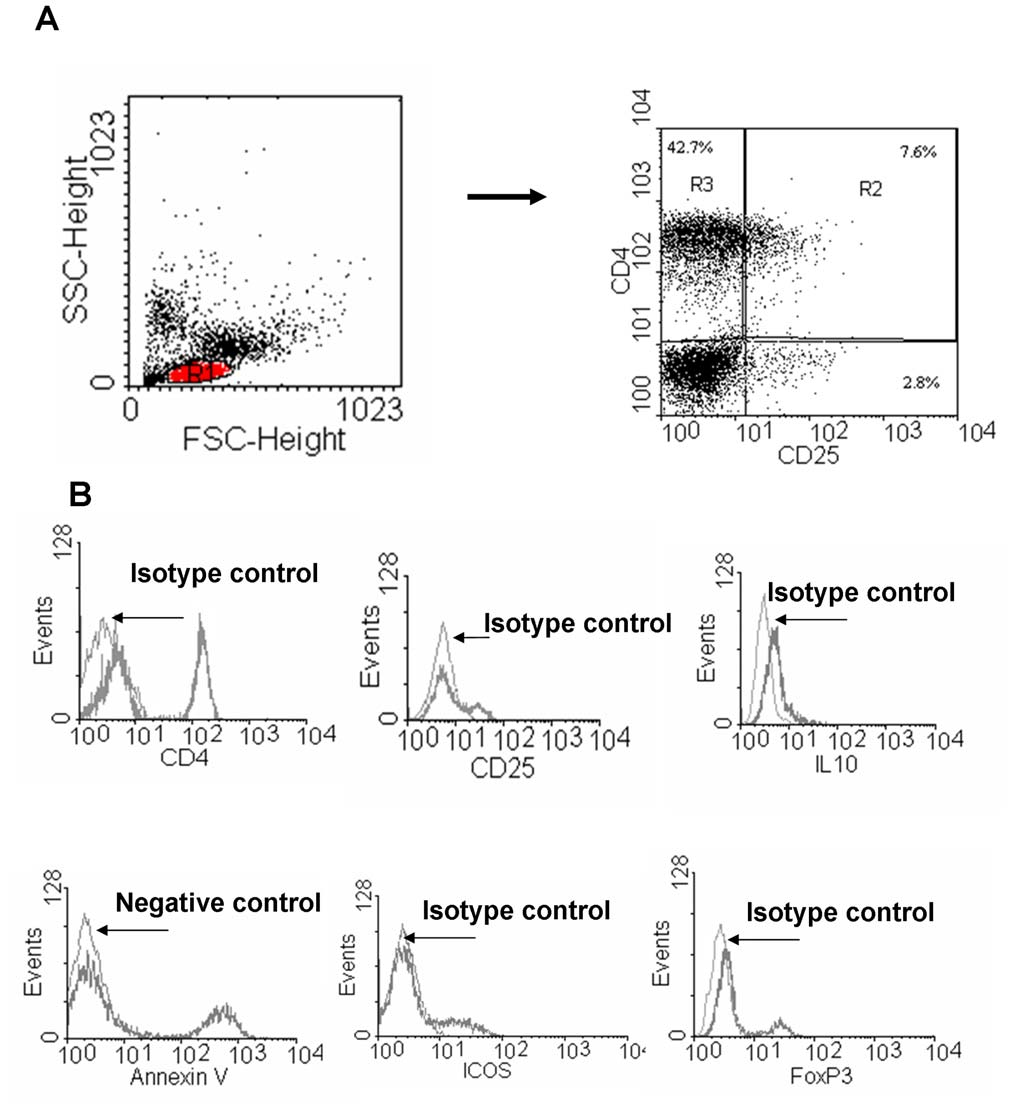
Gating strategy and isotype controls for flow cytometric identification of lymphocyte subpopulations. PBMC from controls (n = 15) and from mild intermittent asthmatics (n = 19) were cultured with/without BUD and assessed for further analyses. The total lymphocytes were gated by forward and side scatter (red coloured R1) and the CD4+CD25− and the CD4CD25+ cells were gated as R 3 and R2, respectively as shown in **A**. Histograms for isotype controls for CD4, CD25, ICOS, IL-10 and Foxp3 staining and negative control for annexin V binding (unstained cells) in total lymphocyte population are shown in **B**.

For flow cytometry, analyses were performed on a Becton Dickinson FACSCalibur System. Lymphocytes were gated by forward and side scatter and negative controls were performed using an isotype control antibody (BD PharMingen) (Figure 1). The analysis, in total lymphocyte gate (R1) was performed on 10,000 events for each sample using CellQuest acquisition and data analysis software (Becton Dickinson).

### Annexin V binding

T cell survival was determined [13] by Annexin V staining in PBMC previously stained with FITC anti-human CD4 and PE-Cy5 anti-human CD25 (BD PharMingen).

PE Annexin V staining was performed using a commercial kit (Bender MedSystem, Vienna, Austria) following the manufacturer's directions. PBMC were analyzed by flow cytometry within 1 hour.

### Expression of surface markers

PBMC were stained with PE anti-human ICOS, with FITC anti-human CD4 and PE-Cy5 anti-human CD25 [all monoclonal antibodies (mAb) were from BD PharMingen].

### Foxp3 intracellular staining

PBMC were stained with PE anti-human CD4 and PE-Cy5 anti-human CD25 (BD PharMingen). Cells were fixed and permeabilized using the BD PharMingen human Foxp3 Buffer Set, following the manufacturer's recommended assay procedure. Finally, the cells were stained with FITC anti-human Foxp3.

### Intracellular cytokine staining

For the detection of intracellular cytokine IL-10, PBMC were cultured overnight with GolgiStop (2 µM final concentration) (BD PharMingen). The cells were stained with CD4 FITC and CD25 PE-Cy5 in PBS containing 1% FCS and 0.1% Na azide for 30 min at 4°C. Cells, washed twice in PBS with 1% FCS, were fixed with PBS containing 4% paraformaldehyde for 20 min at room temperature. After two washes in permeabilization buffer (PBS containing 1% FCS, 0.3% saponin, and 0.1% Na azide) for 15 min at 4°C, the cells were stained with 0.25 µg of PE anti-human IL-10 antibody (BD PharMingen). After two more washes in PBS containing 1% FCS, the cells were analyzed by FACSCalibur.

### Isolation of CD4+CD25+CD127dim Regulatory T cells

CD4+CD25+CD127dim T cells were isolated using magnetic cell sorting (MACS separation columns, Miltenyi Biotec, Germany) kit following the manufacturer's directions as previously described [16].The purified T cells were cultured with or without BUD and then assessed for IL-10 expression by flow cytometry.

### Cell proliferation assay

PBMC were isolated from asthmatic patients (n = 6) and cell proliferation was measured using carboxyfluorescein succinimidyl ester (CFSE) labeling assay. CFSE is used to fluorescently label live cells and is equally partitioned to daughter cells during division [16], [17]. Briefly, the cells were incubated with CFSE (Molecular Probes, Inc. Eugene, OR) (at a final concentration of 5 µM) at 37°C for 10 min. Labeling was blocked by the addition of an equal volume of heat inactivated FCS. Tubes were placed in ice for 5 min and then washed. After centrifugation, the cells, seeded in 24 well plates (1×10^6^ cells/well) in complete RPMI, were stimulated for 24 hours with and without BUD (10^−8^ M final concentration) and then incubated with allergen for 72 hours (37°C- 5% CO_2_). Cell proliferation was assessed by flow-cytometry.

### Statistical analysis

Data are expressed as medians and 25–75 percentiles. All the statistical analyses were performed using the StatView 5.0.1 software. A non-parametric Mann Whitney test for comparisons between the two recruited groups was applied. Statistical analysis of the *in vitro* and *in vivo* effects of BUD was performed by Wilcoxon test P<0.05 was accepted as statistically significant.

## Results

### Demographic characteristics of the subjects

The demographic characteristics, the clinical and functional evaluations and the percentages of peripheral CD4+, CD4+/CD25+ and CD4+/CD25− cells of the studied patients are shown in Table 1. Significantly higher percentages of CD4+ and of CD4+/CD25− cells were observed in both mild intermittent and persistent asthmatics in comparison to controls. No differences were observed between mild intermittent and mild persistent asthmatics.

### In vitro effects of BUD: Annexin V binding in peripheral blood T-lymphocytes

Corticosteroids inhibit T-cell activation and production of Th2 cytokines [10] [11] and increase T cell apoptosis [12] [13]. We initially approached the study assessing *in vitro* the effects of BUD in the apoptosis (annexin V binding) of total lymphocytes, of CD4+/CD25+ and of CD4+/CD25- cells.

**Figure 2:**
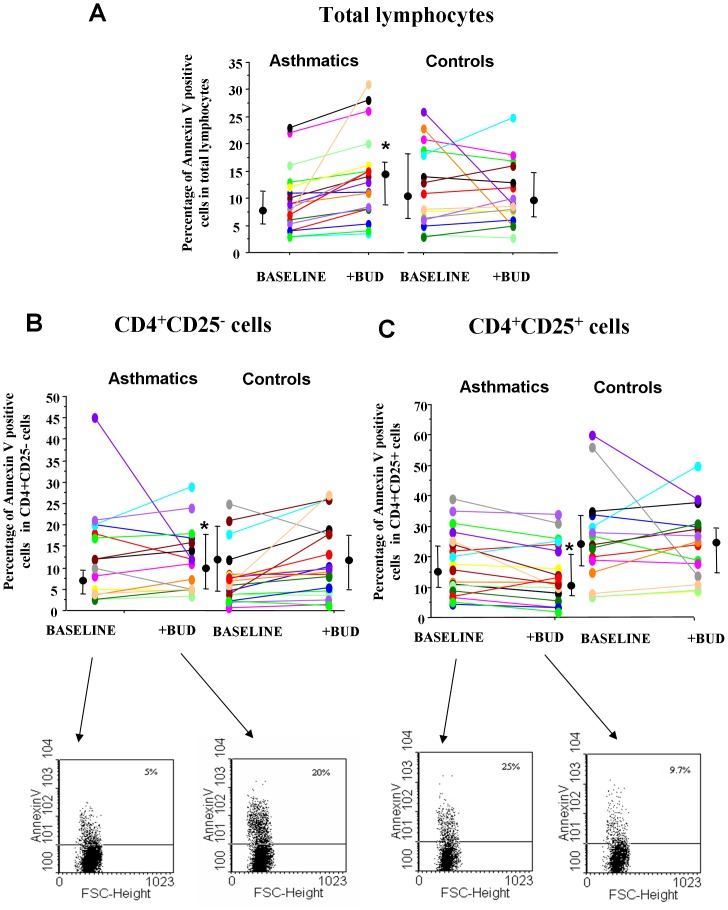
BUD modulates *in vitro* the annexin V binding of peripheral blood T-lymphocytes. PBMC from controls (n = 15) and from mild intermittent asthmatics (n = 19) were cultured with/without BUD and were assessed for annexin V binding in total lymphocytes (**A**), in CD4+CD25− (**B**) and in CD4+CD25+ cells (**C**). Individual results, median and 25–75 percentiles are shown. *p<0.05 baseline vs BUD. Arrows indicate the representative dot plots.

The ability of BUD to increase annexin V binding was assessed in total lymphocytes, in CD4+/CD25+ and in CD4+/CD25- cells. The total lymphocytes, CD4+CD25+ and the CD4CD25- cells were gated as shown in figure 1A–B. No significant differences in the annexin V binding between asthmatics and normal subjects were detected in total lymphocytes or in CD4+/CD25+ and in CD4+/CD25− cells (figure 2 A, B, C). BUD significantly increased the annexin V binding in total lymphocytes (figure 2 A) and in CD4+/CD25− cells (figure 2 B) while it decreased the annexin V binding in CD4+/CD25+ cells in asthmatic patients but not in controls (figure 2 C).

### Expression of ICOS on peripheral blood T-lymphocytes sub-populations

**Figure 3:**
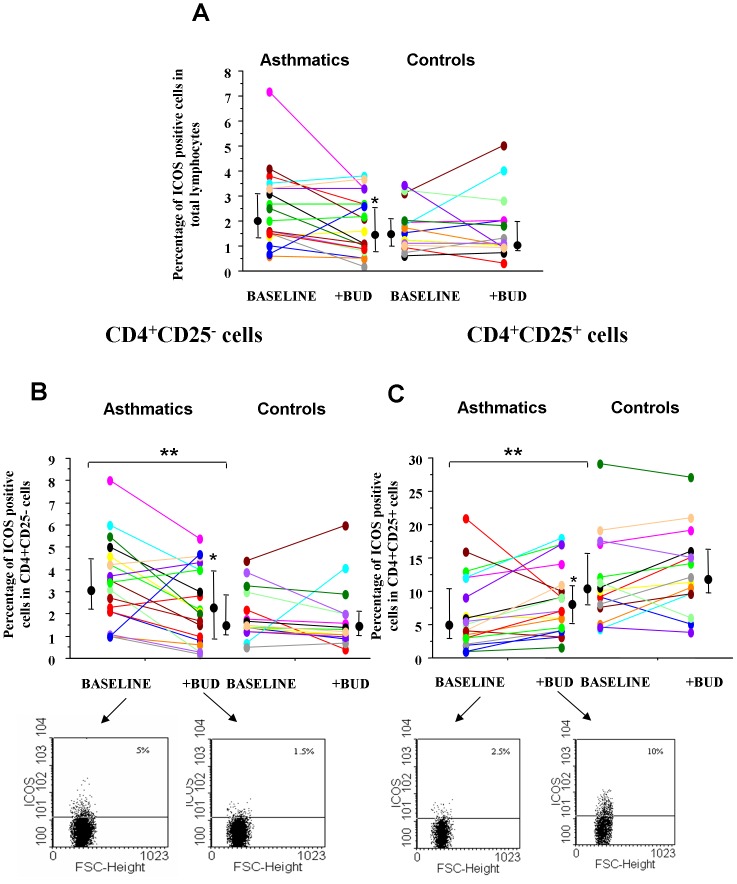
BUD affects *in vitro* the expression of ICOS on peripheral blood T-lymphocytes. PBMC from controls (n = 15) and from mild intermittent asthmatics (n = 19) were cultured with/without BUD and were assessed for ICOS expression by flow-cytometry in total lymphocytes (**A**), in CD4+CD25− cells (**B**) and in CD4+CD25+ (**C**) cells from controls (n = 15) and from asthmatics (n = 19). Individual results, median and 25–75 percentiles are shown.*p<0.05 baseline vs BUD.**p<0.05 asthmatics vs controls. Arrows indicate the representative dot plots.

ICOS is a co-stimulatory molecule acting as an important regulator of Th2 lymphocyte function [18]. The effect of BUD in modulating ICOS expression was assessed in total lymphocytes (figure 3 A), in CD4+/CD25+ (figure 3 B) and in CD4+/CD25− cells (figure 3 C). The percentage of ICOS positive cells was significantly reduced in CD4+/CD25+ cells while it was significantly increased in CD4+/CD25− cells in asthmatics when compared to controls. BUD reduced the percentage of ICOS positive total lymphocytes in asthmatics but not in controls (figure 3 A). In asthmatic patients, BUD significantly reduced the percentage of ICOS positive cells in CD4+/CD25− cells (figure 3 B) while it increased the percentage of ICOS positive cells in CD4+/CD25+ cells (figure 3 C).

### Expression of Foxp3 in peripheral blood T-lymphocyte sub-populations

**Figure 4:**
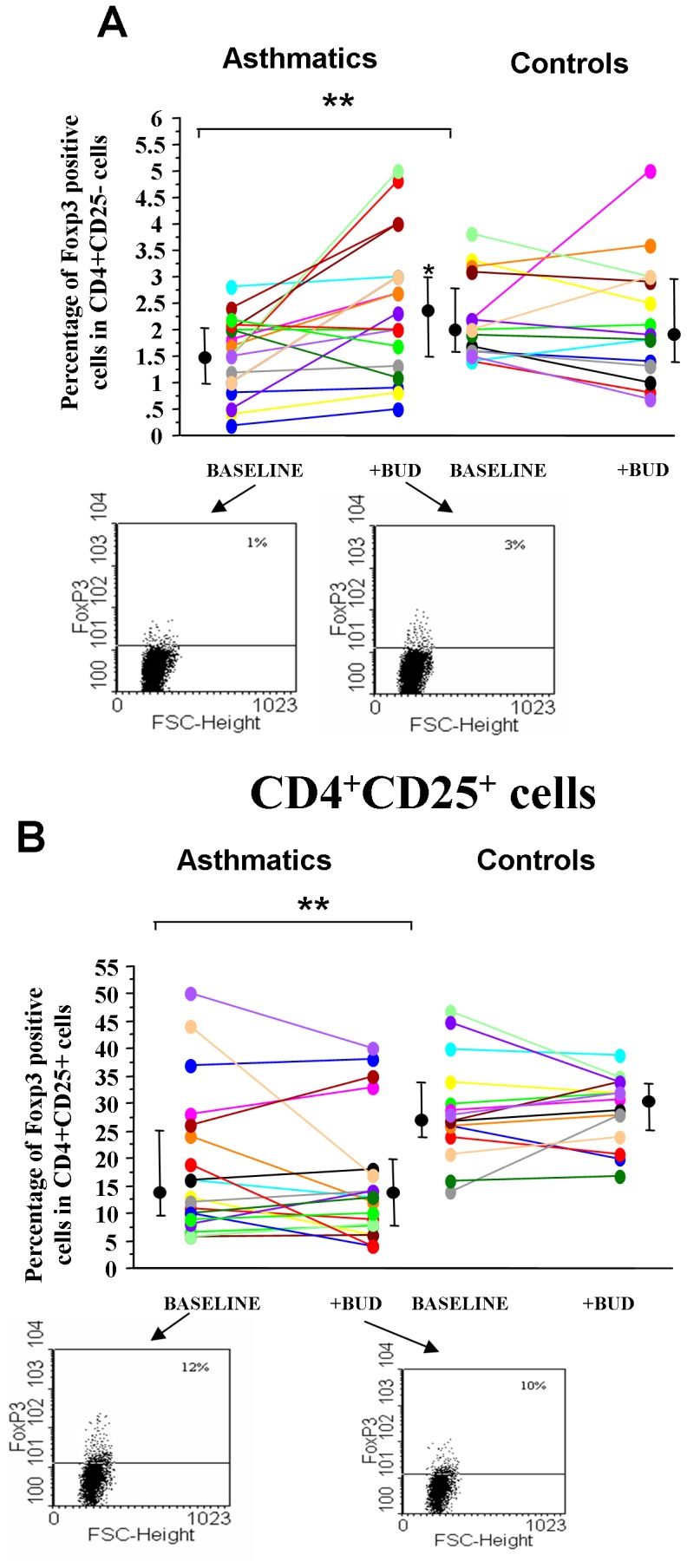
BUD affects *in vitro* the expression of Foxp3 in CD4+CD25+ and CD4+CD25- peripheral blood T-lymphocytes. PBMC were isolated from controls (n = 15) and from mild intermittent asthmatics (n = 19), were cultured with/without BUD and were assessed for Foxp3 by flow-cytometry in CD4+CD25− (**A**) and in CD4+CD25+ cells (**B**). Individual results, median and 25–75 percentiles are shown.*p<0.05 baseline vs BUD.**p<0.05 asthmatics vs controls. Arrows indicate the representative dot plots.

In asthmatics decreased T regulatory activities are observed [7]. The effect of BUD in modulating Foxp3+ expression, a transcription factor characterizing Tregs, was assessed in CD4+/CD25+ and in CD4+/CD25− cells from asthmatics and from controls. A reduced percentage of Foxp3 positive cells was observed in both CD4+/CD25+ and in CD4+/CD25− cells from asthmatics when compared to controls and BUD was able to significantly increase the percentage of Foxp3 positive cells in CD4+/CD25− but not in CD4+/CD25+ cells in asthmatics (figure 4 A–B).

### Expression of IL-10 in peripheral blood T-lymphocytes sub-populations

**Figure 5:**
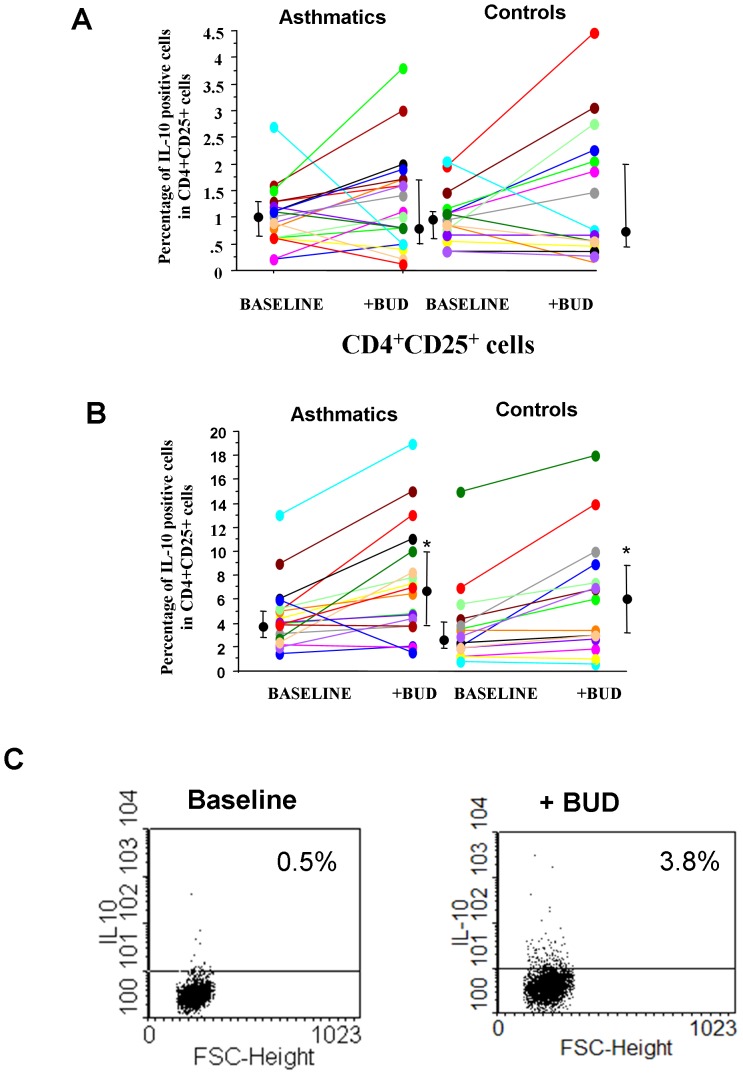
BUD affects *in vitro* the expression of IL-10 in CD4+CD25+. PBMC were isolated from controls (n = 15) and from mild intermittent asthmatics (n = 19), were cultured with/without BUD and were assessed for IL-10 expression by flow-cytometry in CD4+CD25− (**A**) and in CD4+CD25+ cells (**B**). Individual results, median and 25−75 percentiles are shown.*p<0.05 baseline vs BUD. **C.** The expression of IL-10 was also assessed in CD4+CD25+CD127 dim T cells (see materials and methods for details). Representative dot plot of cells from a patient with asthma at baseline and following *in vitro* BUD exposure is shown.

Corticosteroids act on Tregs increasing IL-10 production [9]. The effect of BUD in the expression of IL-10 was assessed in CD4+/CD25− cells (figure 5 A) and in CD4+/CD25+ cells (figure 5 B) from asthmatic patients and from controls. BUD was able to increase the percentage of IL-10 positive cells in CD4+/CD25+ cells (figure 5 B) but not in CD4+CD25− cells (figure 5 A). The effect of BUD in IL-10 expression was also assessed in CD4+CD25+CD127dim T cells to establish whether the observed effects of BUD are related to Treg or to activated effector T cells that can transiently express Foxp3. As shown in figure 4C, BUD was able to increase the expression of IL-10 in CD4+CD25+CD127dim T cells.

### Proliferation of peripheral blood T-lymphocytes

**Figure 6:**
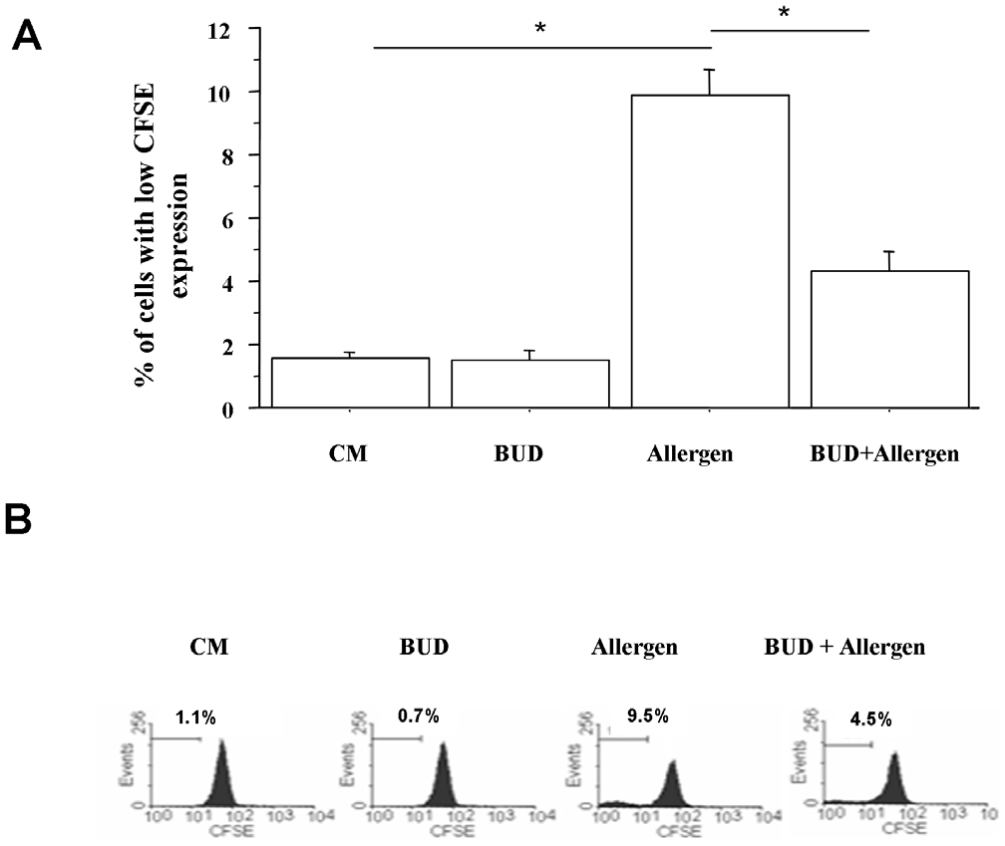
Effects of BUD in peripheral T cell proliferation. For cell proliferation experiments, PBMC were isolated from mild intermittent asthmatics (n = 4), cultured with and without BUD for 24 hours and then stimulated for 72 hours with the most relevant allergen for each patient. Cell proliferation was assessed using CFSE and analysed by means of flow-cytometry. The analysis were performed on lymphocytes gated by forward and side scatter. Data are expressed as % of cells with low CFSE expression (**A**). * p<0.05 (paired t test). Histograms from one representative experiment is shown (**B**).

We tested whether BUD affected the proliferation of T lymphocytes upon allergen exposure. When peripheral T lymphocytes were stimulated with allergen a significantly increased cell proliferation was observed. BUD significantly reduced T lymphocyte proliferation counteracting the effects of the allergen (figure 6).

### In vivo effects of BUD

**Figure 7:**
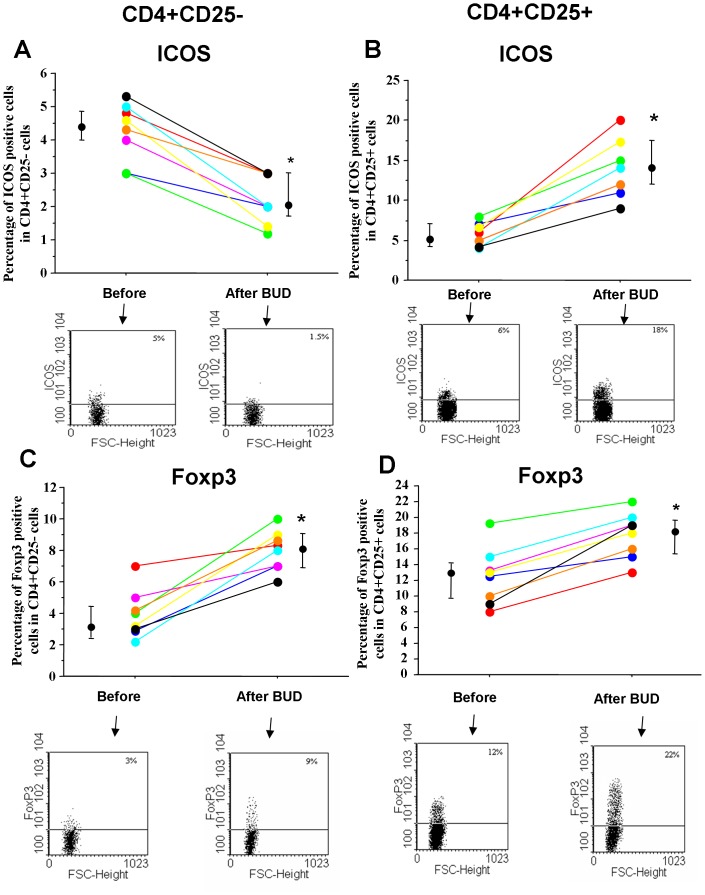
In vivo effects of BUD in ICOS and Foxp3 expression in peripheral blood T-lymphocytes. PBMC were isolated from mild persistent asthmatics (n = 8) before and after inhaled BUD treatment (see Materials and Methods for details). ICOS (**A–B**) and Foxp3 (**C–D**) were assessed by flow-cytometry in CD4+CD25− and in CD4+CD25+ cells. Individual results, median and 25–75 percentiles are shown. *p<0.05. Arrows indicate the representative dot plots.

**Figure 8:**
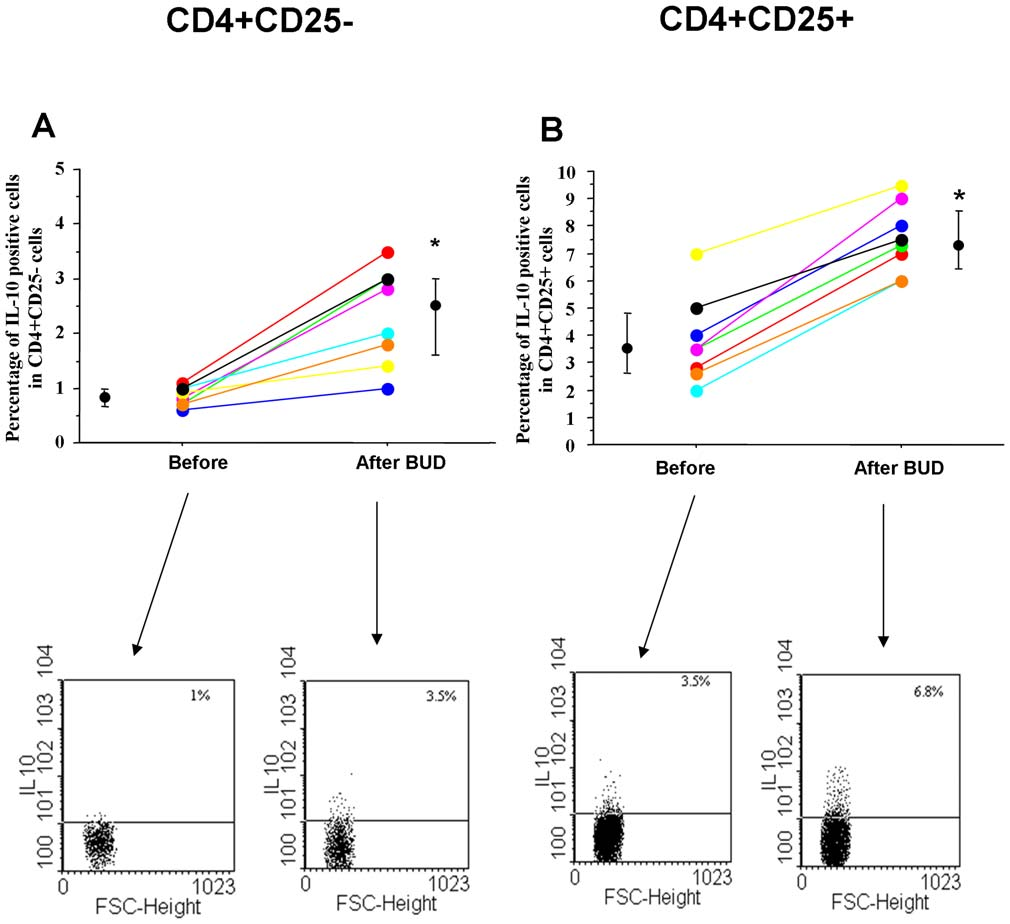
In vivo effects of BUD in IL-10 expression in peripheral blood T-lymphocytes. PBMC were isolated from mild persistent asthmatics (n = 8) before and after inhaled BUD treatment (see Materials and Methods for details). IL-10 expression were assessed by flow-cytometry in CD4+CD25− (**A**) and in CD4+CD25+ (**B**) cells. Individual results, median and 25–75 percentiles are shown. *p<0.05. Arrows indicate the representative dot plots.

Finally, the in vivo effects of BUD were tested in mild persistent asthmatics. BUD was able to increase the percentage of ICOS positive cells in CD4+/CD25+ cells while it decreased the percentage of ICOS positive cells in CD4+/CD25− cells (Figure 7 A–B). Moreover, BUD was able to increase in CD4+CD25+ and in CD4CD25− cells the percentages of Foxp3 (Figure 7 C–D) and IL-10 (Figure 8 A–B) positive cells. After BUD treatment, these immunomodulatory effects paralleled the patient clinical benefits including an improvement of peak flow (Morning PEF (L min^−1^) baseline = 382±40; BUD = 441±38; p<0.02. Evening PEF (L min^−1^) baseline = 421±50; BUD = 460±45; p<0.02), improvements in the basal FEV1 (median increase of 15%), no use of rescue medications, improvement of ACT symptom score (baseline = 15.8±2.2, BUD = 20.7±1.6; p<0.02).

## Discussion

The present study demonstrates that BUD, an inhaled corticosteroid widely used for the management of bronchial asthma, controls asthma inflammation modulating ICOS, cell survival, Foxp3 and IL-10 expression differently in CD4+/CD25− and in CD4+/CD25+ cells in peripheral blood. These mechanisms of action of BUD were demonstrated by an *in vitro* and an *in vivo* approach and provide further understanding on new mechanisms by which corticosteroids control T lymphocyte activation. T-lymphocytes play a crucial role in the development of airway inflammation. Th2 induce and Treg cells suppress several features of allergic inflammation in asthmatics. One of the mechanisms leading to the persistence of T- cell activation in asthma may be related to a reduced T cell survival [19]. T-lymphocytes represent an important target of anti-inflammatory drugs, and particularly of corticosteroids. One of the mechanisms mediated by corticosteroids in target cells is the induction of cell apoptosis [20], a phenomenon that can be assessed by the annexin V binding method. Up to now it is unclear whether the preferential activation of cell apoptosis in specific T lymphocyte subpopulations may explain the therapeutic activities of corticosteroids in asthmatic patients. Only a single Japanese study by Oneda [21] demonstrates that in asthma dexamethasone induces cell apoptosis preferentially in T activated effector cells but no study has explored the effect of BUD in cell survival simultaneously in total lymphocytes, CD4+/CD25+ and CD4+/CD25− cells from asthmatics. In the present study CD4+/CD25+ and CD+/CD25− were not purified to avoid non specific activation and to assess the BUD effects in a microenvironment where, as *in vivo*, mixed populations of immunocompetent cells (including accessory cells) are present.

Here, it is demonstrated that asthmatic patients showed increased percentages of CD4+ and CD4+/CD25− cells and that BUD significantly increased the percentage of annexin V positive cells in total lymphocytes as well as in CD4+/CD25−cells. Differently, BUD decreases the percentage of annexin V positive cells in CD4+/CD25+ cells in asthmatic patients. In this regard it also been demonstrated that a low dose of IL-2 can protect CD4+CD25+ but not CD4+CD25− cells from dexamethasone-induced apoptosis by affecting forkhead box O3a phosphorylation through the Akt and serum and glucocorticoid-induced protein kinase pathways [22].This latter phenomenon may be also due to the fact that Foxp3 highly expressed on a portion of CD4+/CD25+ cells, regulating glucocorticoid-induced TNF receptor expression [23], confers protection from TCR-mediated apoptosis [24].

In asthma, T lymphocyte expansion, other than promoted by increased cell survival [17], may be also promoted by other mechanisms [25]. T lymphocytes to become fully activated require a nonspecific co-stimulatory signal. ICOS, a co-stimulatory molecule, is up-regulated after T lymphocyte activation and is retained on many memory T lymphocytes. This molecule is preferentially expressed on Th2 cells and plays an important role in the production of IL-2, IL-4, and IL-5 from recently activated T lymphocytes [3]. ICOS is also expressed by Tregs and exerts a fundamental role in the generation and function of CD4+CD25+Foxp3+ regulatory T cells [26]. ICOS-ICOS-ligand interactions and IL-10 regulate the development and the inhibitory function of regulatory lymphocytes [27]. Tregs with high ICOS expression mediate stronger suppression activities [28]. In the present study, we demonstrate for the first time that asthmatic patients show a reduced percentage of ICOS positive cells in CD4+/CD25+ cells while they show increased percentage of ICOS positive cells in total lymphocytes and in CD4+/CD25− cells when compared to controls. BUD reduced percentage of ICOS positive cells in total lymphocytes and in CD4+/CD25− cells in asthmatic patients, contributing to limit the activation of these cells. Accordingly, it has been demonstrated that dexamethasone reduces allostimulatory properties inhibiting the expression of co-stimulatory molecules on dendritic cells [29]. Moreover, prednisolone reduces the serum concentrations of a co-stimulatory ligand, sCD86 in allergic asthma [30]. Furthermore, we show here that BUD increases ICOS in CD4+/CD25+ further supporting the concept that this drug is effective in controlling T lymphocyte activation also improving the function and the activities of the Tregs. T regulatory activities are manly related to naturally occurring CD4+/CD25+ Tregs and to CD4+/CD25− inducible Tregs [31]. High percentages of CD4+/CD25+ Tregs express Foxp3, a transcription factor typically associated to T regulatory activities [32]. This natural CD4+/CD25+ subset is thymus-born, constitutively expresses IL-10 mRNA, does not produce IL-2 and is resistant to apoptosis [32].

T regulatory activities have the potential to suppress pathogenic Th2 responses thus preserving lung integrity [33] and may be defective or overridden in patients with allergic diseases including asthma [7]. The levels of Foxp3 mRNA in BAL from children with asthma are lower than in healthy controls, positively correlate with FEV1 and, after 4 weeks of treatment with inhaled corticosteroids, significantly increase [34]. Furthermore, in severe asthmatics, the Foxp3 protein expression in PBMC correlate with the FEV1 values and the symptom score [35].

In the present study we show that a reduced percentage of Foxp3 positive cells was observed in CD4+/CD25+ and in CD4+/CD25− cells from asthmatics when compared to the controls. BUD *in vitro* is able to significantly increase the percentage of Foxp3 positive cells in CD4+CD25−. This is the first time that this effect of a corticosteroid is demonstrated in the CD4+/CD25− cell population and this phenomenon may be relevant in the control of bronchial inflammation since CD4+/CD25− T lymphocytes ectopically expressing Foxp3 acquire the function to control inflammation in experimentally induced inflammatory bowel disease and to down-regulate the proliferation of CD4+/CD25− T lymphocytes in vitro [36]. Although a previous report in patients with systemic lupus erythematosus shows that dexamethasone enhances Foxp3 expression without inducing a higher antiproliferative function [37], we demonstrate here that BUD reduced T lymphocyte proliferation upon allergen stimulation in allergic asthmatics. Tregs control effector immune responses through a diverse array of mechanisms including secretion of the anti-inflammatory cytokines TGF and IL-10 [38]. IL-10 is essential not only for suppression of effector cells by Treg cells but also for their differentiation [27]. IL-10, produced by a number of different cells, affecting antigen presenting cell function, dendritic cell maturation as well as the activation of co-stimulatory molecules, can inhibit both Th1 and Th2 type responses [9]. IL- 10 activates tyrosin phosphatase 1 which dephosphorylates rapidly CD28 and ICOS co-stimulatory receptors [39]. Immunosoppressive drugs induce IL-10 producing cells from naive CD4+/CD25− cells [9] and in the present study BUD was able to increase the expression of IL-10 in CD4+/CD25+ cells and in CD4+/CD25+/CD127 dim T cells. The reduced expression of CD127 distinguish cells that are activated effector cells (that also increase CD25 and Foxp3, albeit transiently) from genuine CD4+CD25+Foxp3+ Treg [40]. Rapid switch and expansion of IL-10-producing cells and the use of multiple suppressive factors represent essential mechanisms in immune tolerance to a high dose of allergens in non-allergic individuals [41]. In the present study inhaled BUD in patients with mild persistent asthma is able, *in vivo*, to increase ICOS, Foxp3 and IL-10 expression in CD4+/CD25+ while it decreased ICOS expression and increased Foxp3 and IL-10 expression in CD4+/CD25− cells. In these patients these immuno-regulatory events promoted by BUD treatment are associated to clinical benefits including improvements of FEV1, stability of peak flow values and absence of symptoms. Furthermore, additional studies on lymphocytes isolated from bronchoalveolar lavages will allow to confirm these immuno-regulatory effects of BUD also within the lower respiratory tract.

In conclusion, these results suggest that BUD is effective in controlling asthma inflammation by reducing both T lymphocyte survival and T lymphocyte co-stimulatory ICOS in CD4+/CD25− and by increasing the survival and the activities of CD4+/CD25+ cells. These mechanisms described i*n vitro* are translated in vivo and may contribute to improve asthma control. Future studies are needed to clarify whether these immunomodulatory effects of BUD are shared by other inhaled steroids.

## Supporting Information

Figure S1 **PBMC were cultured with different concentrations of BUD (10^−7^, 10^−8^ and 10^−9^ M) and after selecting the best concentration of BUD (10^−8^ M) two time points (24 and 48 hrs) were tested.** Representative dot plots were shown.
(TIF)
