# Expression of Concern: Multiple In Vitro and In Vivo Regulatory Effects of Budesonide in CD4+ T Lymphocyte Subpopulations of Allergic Asthmatics

**DOI:** 10.1371/journal.pone.0312566

**Published:** 2024-10-18

**Authors:** The PLOS ONE Editors

Following publication of this article [1] concerns were raised regarding the results presented in Figs 2–4. Specifically:

- In Figs 2–4 the following panels containing FACS data appear more similar than would be expected from independent results:
  ○ The asthmatic + budesonide panel in Fig 2B and the asthmatic baseline panel in Fig 2C.
  ○ The asthmatic + budesonide panel in Fig 3B and the asthmatic baseline panel in Fig 3C.
  ○ The asthmatic baseline panel and the asthmatic + budesonide panel in Fig 4A.
  ○ The asthmatic baseline panel and the asthmatic + budesonide panel in Fig 4B.

The corresponding author stated that the similarities in the above panels are due to stimulation of the same cell population from the individual donor represented in the respective panels, and provided additional data from other participants within the study. However, the authors stated that they were unable to share the original FACS data underlying this study, as these files contain sensitive patient data, and the authors did not receive permission from the CNR Data Protection Officer to share these data publicly or for editorial review only.

PLOS remains concerned that the panels listed above appear more similar than would be expected from independent samples, and we do not consider that replacement data alone are sufficient to resolve the concerns raised with the published figures. In the absence of the original underlying data, the concerns raised with this article cannot be fully resolved.

The *PLOS ONE* Editors issue this Expression of Concern to inform readers of the above concerns, which cannot be fully resolved in the absence of the original underlying data, and recommend that readers interpret these results with caution.
